# Compressibility and Anisotropy of Trona: Unveiling the Structure of a Dense Na_3_H(CO_3_)_2_·2H_2_O Polymorph

**DOI:** 10.1021/acs.inorgchem.5c00642

**Published:** 2025-05-15

**Authors:** Benedito Donizeti Botan-Neto, David Santamaria-Perez, Lena Wedek, Lkhamsuren Bayarjargal, Ganesh Bera, Pablo Botella, Julio Pellicer-Porres, Alberto Otero-de-la-Roza, Catalin Popescu, Frederico G. Alabarse

**Affiliations:** † Departamento de Física Aplicada-ICMUV, MALTA Consolider Team, Universitat de València, Valencia 46100, Spain; ‡ Institute of Geosciences, 9173 Goethe University Frankfurt, Frankfurt 60438, Germany; § Departamento de Química Física y Analítica, Facultad de Química, MALTA Consolider Team, Universidad de Oviedo, Oviedo 33006, Spain; ∥ CELLS-ALBA Synchrotron Light Facility, Cerdanyola del Vallés, 08290 Barcelona, Spain; ⊥ Elettra Sincrotrone Trieste, Trieste 34149, Italy

## Abstract

Understanding the structural stability of hydrated carbonates and bicarbonates under different thermodynamic conditions is crucial, as they play an important role in the carbon cycle, in environmental chemistry, and geochemistry. Sodium sesquicarbonate dihydrate, Na_3_H­(CO_3_)_2_·2H_2_O trona, is a naturally occurring evaporite mineral also found in magmatic environments. In this work, we carried out high-pressure (HP) in situ synchrotron powder and single-crystal X-ray diffraction (XRD) measurements on a naturally occurring trona specimen. A high-pressure monoclinic to triclinic phase transition occurred at 12.8 GPa, and the structure of the dense HP trona phase was solved. The coordination number of sodium atoms increased from 6 in the low-pressure polymorph to 7–8 in the HP one. Additionally, our results did not show any indication of the phase transition previously reported at 7 GPa based on Raman spectroscopy. The compressibility and anisotropy of low- and high-pressure phases were determined. Birch–Murnaghan equation of state parameters were fitted to the pressure–volume data sets, yielding a bulk modulus of 35(2) GPa (*K*′ = 4.4(9)) for trona using neon as pressure-transmitting medium. Density functional theory calculations supported the experimental results, confirming the structural stability of the phases obtained by XRD.

## Graphical abstract

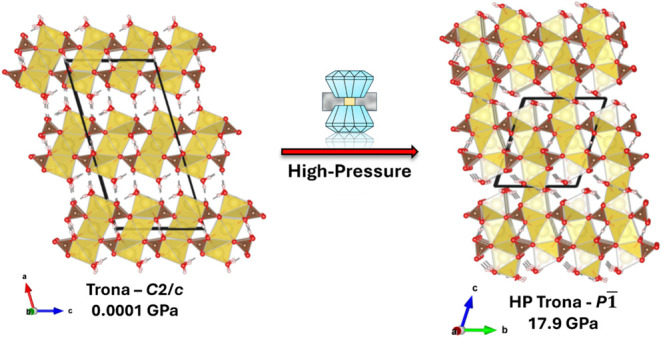

## Introduction

Hydrated carbonates and bicarbonates are compounds that play a crucial role in various natural and industrial processes, often serving as important intermediaries in the carbon cycle, in environmental chemistry, and geochemistry. In nature, hydrated bicarbonates are found in sedimentary deposits and serve as reservoirs for carbon.^1,2^ They are involved in buffering systems in aquatic environments, where they help maintain the pH balance of oceans and freshwater bodies, playing a pivotal role in regulating atmospheric CO_2_ levels.^3^ In addition, they are central to processes like mineral precipitation and dissolution, which influence soil formation.^4^ In an industrial context, carbonates and bicarbonates are part of processes such as the production of glass, paper, and chemicals.^5^ Sodium carbonate, often in hydrated forms, is widely used in the manufacture of detergents.^6^ Its hydrated bicarbonates are also key, for instance, in baking, fire extinguishing, and as mild abrasives.^7^ The physical properties and chemical behavior of these compounds intimately relates to the presence of water molecules, which significantly affects their lattice structure. Thus, the structural characteristics of these compounds, particularly the coordination of water molecules and the arrangement of carbonate or bicarbonate anions, are key to understanding their stability, reactivity, and interactions in different environments.

Trona, Na_3_H­(CO_3_)_2_·2H_2_O, is a naturally occurring evaporite mineral which has also been found in magmatic environments, formed by reactions of late-magmatic fluids.^4,8,9^ This compound has significant environmental importance as a product of carbon sequestration of flue gases^10^ and economic importance as a source of soda ash and industrial commodity for numerous manufacturing processes. Several single-crystal X-ray diffraction (XRD) and neutron diffraction studies on Na_3_H­(CO_3_)_2_·2H_2_O have been published.^9,11–15^ At room conditions, trona crystallizes in a *C*2/*c* monoclinic space group and its structure consists of triple chains formed by units of three 6-fold-coordinated edge-sharing sodium polyhedra (an octahedron flanked by two distorted trigonal prisms), as depicted in Figure 1, following the structural model reported by O’Bannon et al.^9^ These chains are linked via carbonate groups in one direction and via H-bonds in the other. In addition, two carbonate groups are hydrogen-bonded to a common H atom, forming a complex [CO_3_–H···CO_3_]^3–^ unit. According to the literature, this H atom is dynamically disordered between two equivalent sites separated by distances that range between 0.211(9) Å^14^ to 0.95(4) Å,^15^ pointing to the existence of a bicarbonate [HCO_3_]^−^ group. At room pressure, trona begins to decompose at ∼348 K.^16^ A pioneering study of the stability of the compound at different thermodynamic conditions was done by Liu and Fleet,^17^ who reported an enhancement of the thermal stability with increasing pressure. At only 2.1 kbar, in equilibrium with CO_2_-rich vapor, trona is stable up to 848 K. Later, O’Bannon et al.^9^ studied the interplay between the trona crystal structure and its stability at (i) high pressure (up to 25.4 GPa) and room temperature using infrared (IR) and Raman spectroscopy measurements and (ii) room pressure and low temperature (down to 100 K) using XRD measurements. The authors found discontinuous and reversible changes in the IR and Raman spectra at ∼7 and ∼14.5 GPa which they tentatively associated with a shift in the sodium–oxygen polyhedra and a change in the coordination geometry of these Na–O polyhedra, respectively. The nature of the structural changes upon compression were, however, not determined, including the structures of the dense polymorphs.

**Figure 1:**
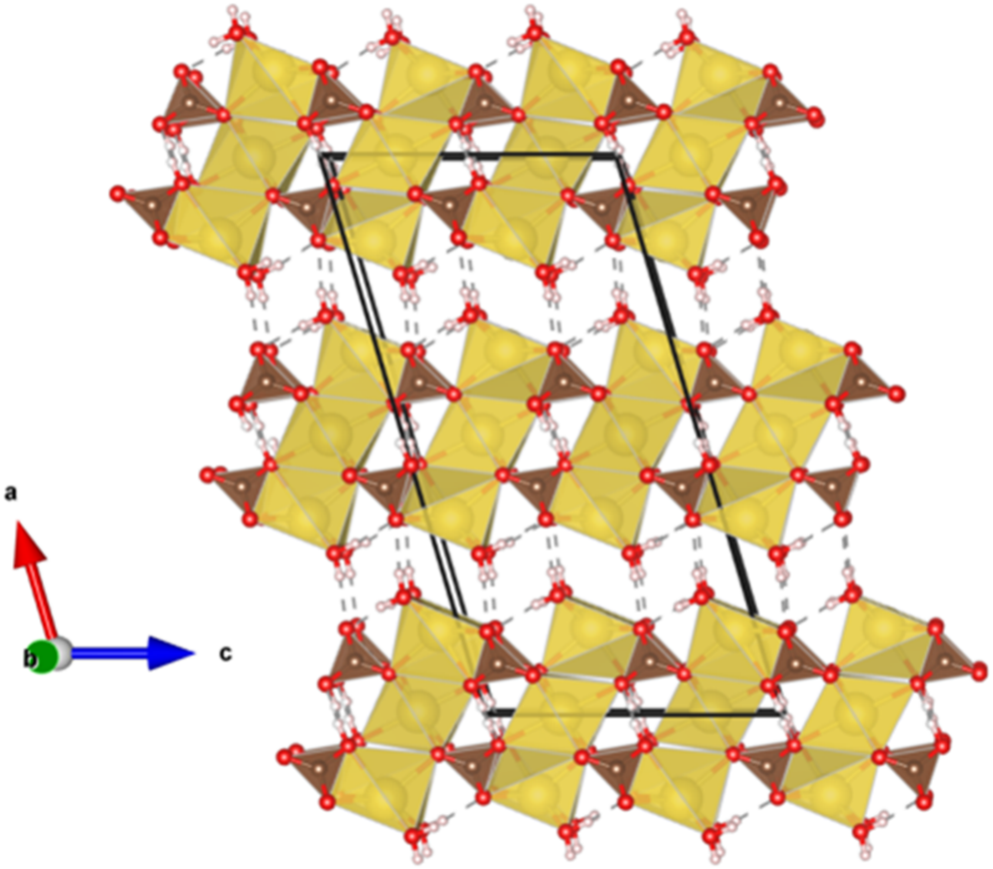
Projection of Na_3_H­(CO_3_)_2_·2H_2_O trona along the *b*-axis at room conditions. Sodium (Na), oxygen (O), carbon (C), and hydrogen (H) atoms are represented in yellow, red, brown, and white, respectively.

In this work we have studied the compressibility and anisotropy of the initial Na_3_H­(CO_3_)_2_·2H_2_O trona mineral using different quasi-hydrostatic pressure-transmitting media. Our XRD measurements do not show considerable structural changes below 12.8 GPa, providing no indication of the phase transition previously reported at 7 GPa based on Raman spectroscopy.^9^ Additionally, we determined by means of in situ synchrotron microfocus single-crystal X-ray diffraction the structure of the unknown denser high-pressure (HP) phase after compressing trona at 14.3 and 17.9 GPa. The compressibility and anisotropy of the two polymorphs were also determined. DFT calculations complement the experimental results. A detailed discussion of the evolution of the atomic arrangement of the trona phases is provided.

## Experimental Details

A natural Na_3_H­(CO_3_)_2_·2H_2_O trona sample from Searles Lake, California, was obtained from Gunnar Färber Mineralien. Chemical analyses were performed on a Philips XL30 scanning electron microscope using energy-dispersive X-ray spectroscopy (EDS). EDS measurements detected the presence of Na, C, and O, consistent with the ideal trona composition, with a Na/O wt % ratio of 0.50(4), which is close to the theoretical ratio of 0.54. Additionally, XRD measurements under room conditions confirmed the trona structure.^14^

Single-crystal XRD was performed at the PETRA III synchrotron (DESY) in Hamburg, Germany, using the Extreme Conditions Beamline P02.2.^18^ A monochromatic X-ray beam with a wavelength of 0.2912 Å, focused by Kirkpatrick Baez (KB) mirrors producing a spot size of 2 μm in diameter, was employed for the experiments. Diffraction patterns were acquired using a PerkinElmer XRD1621 detector with a detector-to-sample distance of 405 mm and a cerium oxide (CeO_2_) standard was used for calibration. A Boehler-Almax-type diamond-anvil cell (DAC) with an 85° aperture angle and diamond culets of 350 μm was employed.^19^ A trona single crystal (30 × 20 × 15 μm) was loaded into a 150-μm-diameter hole of a rhenium gasket preindented to a thickness of 60 μm. A ruby chip was placed in the chamber to determine the pressure by the fluorescence method, using the CNSS05 calibration scale.^20^ Helium, used as the pressure-transmitting medium (PTM), was loaded into the DAC using the Geoscience Institute Frankfurt gas loading apparatus, providing a fluid environment up to 11.6 GPa at room temperature^21^ and quasi-hydrostatic medium below 30 GPa.^22^ The DAC was rotated up to ±39° around the axes perpendicular to the beam. Frames were collected in 0.5° steps with a 1 s acquisition time. Following measurement, the reflections were indexed and integrated using the CrysAlis^Pro^ software version 171.43.131a.^23^ The instrumental parameters were calibrated using a single crystal of enstatite (MgSiO_3_), and the resulting calibration file was generated accordingly. The subsequent structural solution and refinement were carried out by Olex2,^24^ which used SHELXT^25^ and SHELXL^26^ for crystal structure determination and refinement, respectively. During compression, the single crystal fragmented into several domains. As a result, the employment of the Domain Auto Finder (DAFi)^27^ to sort a single crystalline domain was necessary. The structure determination for trona’s polymorphs was performed using a minimum resolution of 0.6 Å. To refine the hydrogen atoms of the water molecule, the O–H bond distance was restrained to 0.95(5) Å, with an H–O–H angle of 104.5°, resulting in a restrained intramolecular H–H distance of 1.4(1) Å. All non-hydrogen atoms were refined anisotropically, while hydrogen atoms were refined isotropically. The equivalent isotropic displacement parameters (*U*_eq_) of the hydrogen atoms were constrained to 1.2 times the *U*_eq_ value of the oxygen atoms to which they are bonded. Finally, the obtained structures were submitted to FINDSYM to identify the space-group symmetry and provide the lattice parameters and Wyckoff positions of the atoms in their standard setting.^28^

Two distinct angle-dispersive powder HP-XRD experiments were conducted at room temperature. The first experiment was performed at the Materials Science and Powder Diffraction (MSPD) beamline of the ALBA-CELLS Synchrotron Light Source.^29^ A monochromatic X-ray beam with a wavelength of 0.4246 Å, focused by KB mirrors producing a spot size of 20 μm in diameter, was employed for the experiments. The measurements were performed using a membrane-type DAC with diamond culets of 500 μm. Trona powder was loaded into a 200-μm-diameter hole in a stainless-steel gasket, which was preindented to a thickness of 50 μm. Silicone oil Dow Corning 200 (Sigma-Aldrich) was used as PTM, providing a quasi-hydrostatic environment that ensures relatively small nonhydrostatic stresses up to 10.5 GPa.^22^ Diffraction patterns were acquired using a Rayonix charge-coupled device (CCD) detector with a detector-to-sample distance of 340 mm. The second experiment was conducted at the Xpress beamline at the Elettra Synchrotron Radiation facility.^30^ A monochromatic X-ray beam with a wavelength of 0.4956 Å, focused by a toroidal Pt-coated mirror producing a spot size of 80 μm in diameter, was employed for the experiments. The measurements were performed using a membrane-type DAC with diamond culets of 500 μm. Trona powder was loaded into a 250-μm-diameter hole in a rhenium (Re) gasket, which was preindented to a thickness of 50 μm. Neon (Ne), used as the PTM, was loaded into the DAC using a Sanchez Technologies gas loading apparatus, providing a fluid environment up to 4.7 GPa^21^ at room temperature and a quasi-hydrostatic medium below 15 GPa.^22^ Diffraction patterns were acquired using a DECTRIS PILATUS 3S 6 M detector with a detector-to-sample distance of 550 mm. Both experiments used a lanthanum hexaboride (LaB_6_) standard for calibration, and pressure was measured using the copper (Cu) equation of state (EoS).^31,32^

Detector calibration and integration into conventional 2θ-intensity powder XRD data were carried out using DIOPTAS software.^33^ The indexing of the powder patterns was performed using the UnitCell,^34^ PowderCell,^35^ and FullProf^36^ program packages.

### Computational Details

Density functional theory (DFT) was used to calculate the EoS and geometry of the trona mineral under pressure. We used the projector-augmented wave (PAW) method^37^ implemented in version 6.5 of the Quantum ESPRESSO suite^38^ with data sets from the pslibrary^39^ and the PBEsol functional.^40^ The number of valence electrons were 4 (C), 1 (H), 9 (Na), and 6 (O). The cutoffs for the plane-wave expansion of the Kohn–Sham states and the density were 80 and 800 Ry, respectively. The *k*-point grids for each phase were selected to ensure energy convergence within 0.1 mRy. These grids are 1 × 5 × 2 for trona, 6 × 2 × 2 for the HP phase, and 9 × 3 × 3 and 6 × 2 × 2 for HPT1 and HPT2, respectively, which correspond to tentative structures for stable high-pressure phases. The HPT nomenclature was adopted solely to facilitate referencing the DFT calculations.

In the room-pressure phase, there is disorder related to the hydrogen atom positions involved in hydrogen bonding interactions between bicarbonate and carbonate moieties. We explored all possible configurations of these hydrogens to select the most energetically stable for the subsequent DFT calculations. For all phases, geometry relaxations were carried out at zero pressure, 30 GPa, and, for specific structures, at 14.3 and 17.9 GPa to compare with experimental diffraction data. All geometry relaxations used convergence thresholds of 10^–3^ Ry/bohr in the forces and 10^–4^ Ry in the energies.

The calculated volumes at zero pressure and 30 GPa were also used to set up a uniformly spaced volume grid for the EOS calculation. Constant-volume geometry relaxations were conducted at each point in the grid for every phase. The resulting energy-volume data was used to fit an average strain polynomial EOS with the gibbs2 program,^41,42^ which was also used to calculate the thermodynamic stability under pressure and the transition pressures.

## Results

Static high-pressure techniques provide an excellent tool to modify the atomic interactions in solids, while allowing in situ characterization.^43^ Interatomic and intermolecular interactions become highly repulsive under compression and phase transitions occur to minimize the overall free energy of the system.^44,45^ Using DACs, we have studied the pressure dependent structural properties of Na_3_H­(CO_3_)_2_·2H_2_O trona up to 17.9 GPa at room temperature.

Figures 2 and 3 present the powder XRD patterns of trona during compression using silicone oil and Ne as PTM. Le Bail fits were performed to account for each reflection in the patterns, demonstrating that the trona structure remains stable up to 12.2 GPa in both silicone oil and neon data sets. Beyond this pressure, data collection using neon was not possible due to experimental limitations—specifically, at 8.1 GPa, the intensities of rhenium and copper peaks became stronger than those of the sample, due to the closing of the gasket hole and the beam size. In the silicone oil data set, phase coexistence between the low-pressure and HP phase of trona is observed between 12.8 and 14.7 GPa, evidenced by the appearance of new peaks from the HP phase at approximately 2θ = 2.47, 6.05, and 6.51°. However, the low symmetry of the emerging structure and the nonhydrostatic conditions introduced by silicone oil at these pressures prevented a satisfactory refinement of the pattern. Above 14.7 GPa, the HP phase becomes dominant, with XRD patterns at 16.7 GPa indicating a single-phase structure. Of the two trona phase transitions reported by O’Bannon et al.^9^ at 7 and 14.5 GPa, only the second one could be confirmed in this study (see details of the analyses of single-crystal diffraction data below). As shown in Figure 2, the refinement at 7.4 GPa was performed solely to demonstrate the good agreement between the inital crystal lattice and the powder XRD data, further supporting—based on XRD evidence—the absence of a phase transition at this pressure.

**Figure 2:**
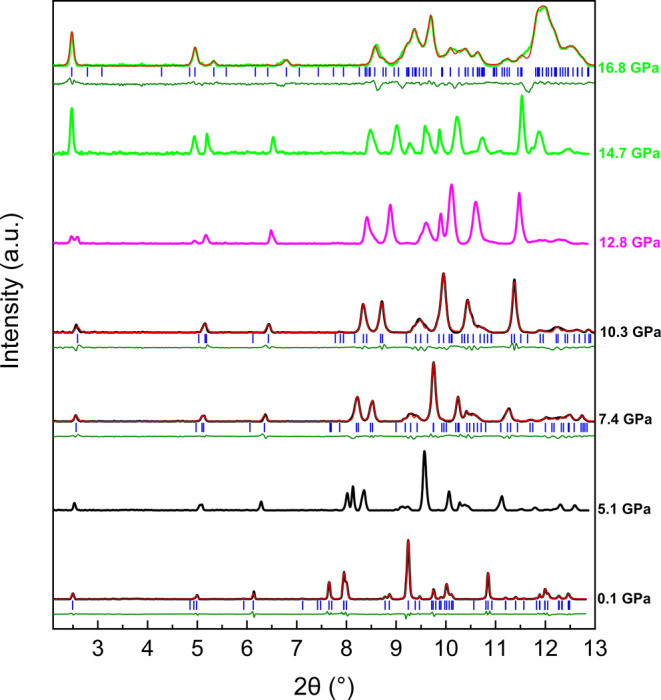
High-pressure XRD patterns of trona up to 16.8 GPa, using silicone oil as PTM. Backgrounds have been subtracted. The black, magenta, and green solid lines represent trona, phase coexistence, and the HP phase patterns, respectively. The red and olive solid lines correspond to the Le Bail fit and the difference profiles, while blue sticks indicate the Bragg positions for the respective phase at the given pressure.

**Figure 3:**
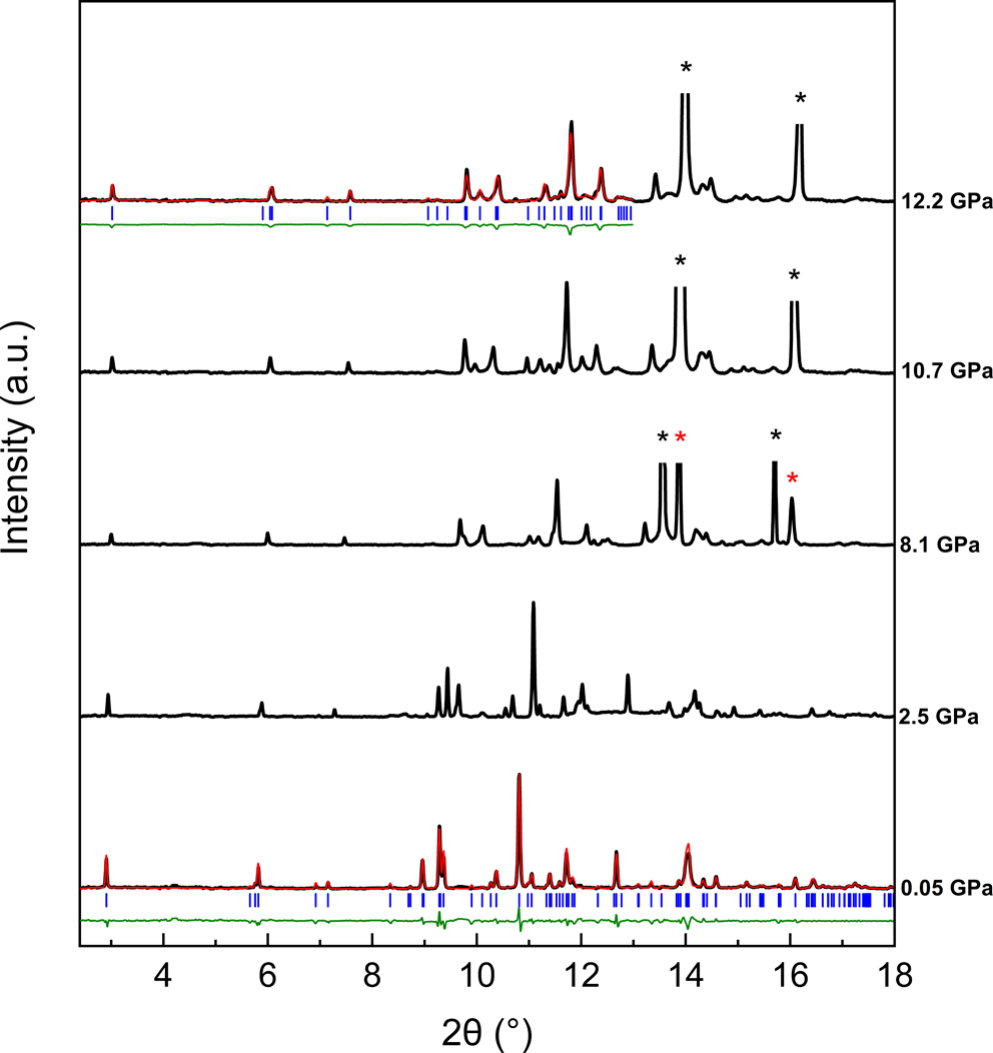
High-pressure XRD patterns of trona up to 12.2 GPa using neon as PTM. Backgrounds have been subtracted. The black, red, and olive solid lines represent the trona, Le Bail fit, and difference profiles, respectively, while blue sticks indicate the Bragg positions for the corresponding phase at each pressure. Black and red asterisks correspond to neon and copper peaks, respectively.

Powder diffraction data presents intensities that do not correspond to perfect randomly oriented powder, so only peak positions and not relative intensities could be used to the structural analysis. In other words, from powder diffraction data, we could only accurately infer the lattice parameters of the mineral upon compression. Indexations of the powder XRD patterns up to 12.2 GPa suggest that the structure can be described by the initial *C*2/*c* monoclinic space group and comparable unit cell dimensions in the whole pressure range. Figure 4 depicts the evolution of trona’s unit cell volume as a function of pressure for two different PTMs: silicone oil (black squares) and neon (red circles), with DFT calculations represented by the green solid line. The data confirms the absence of phase transitions during trona’s compression up to 12.2 GPa. Trona’s compressibility was determined by fitting the Birch–Murnaghan (BM) EoS to the experimental data using the EoSFit7-GUI software.^46^ The third-order BM EoS (BM3) yielded the zero-pressure volume (*V*_0_), bulk modulus (*K*_0_), and first pressure derivative (*K*_p_), while for the second-order BM EoS (BM2), *K*_p_ was implied to be 4. Table 1 summarizes the fitted parameters for experimental and DFT data. The EoS for silicone oil as the PTM was fitted up to 4.6 GPa, below its phase transition at approximately 6 GPa.^22,47^ In contrast, the EoS using neon as the PTM was obtained for the entire pressure range, the unit cell volumes decreasing smoothly upon compression. O’Bannon et al. estimated a *K*_0_ of 36 GPa from P- and S-wave velocities,^9^ which aligns perfectly with the trona EoS fitted using neon as the PTM that yields *K*_0_ values of 36.1(6) and 35(2) GPa for BM2 and BM3, respectively. These values are also in strong agreement with DFT calculations, which suggest a BM3 EoS for trona with *K*_0_ of 35.6 GPa and *K*_p_ of 3.9, allowing the data to be reasonably fitted using a BM2 model as well. On the other hand, the data using silicone oil as the PTM results in *K*_0_ values of 43.2(11) and 39(4) GPa for BM2 and BM3, respectively, highlighting the influence of the lack of hydrostaticity of the PTM on the fitted parameters.^48,49^ The effects of hydrostaticity presented in this work could explain why our data do not coincide with the previously reported phase transition of trona at 7 GPa, which may have resulted from the use of a nonhydrostatic PTM such as KBr.^9^

**Figure 4:**
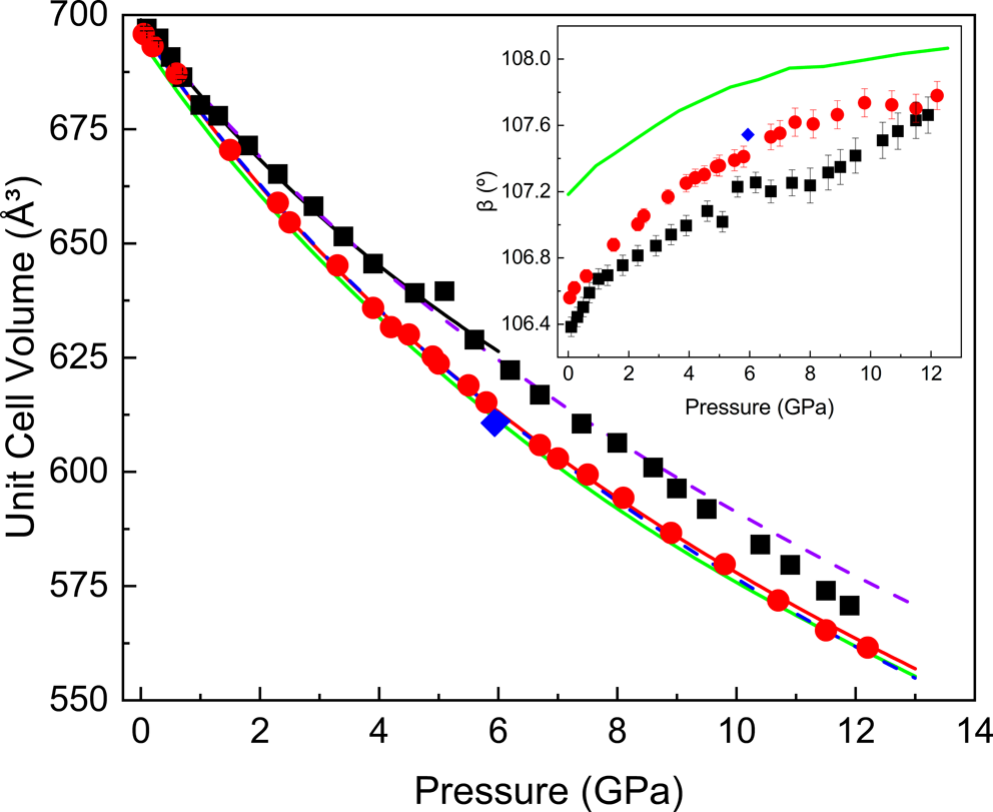
Pressure dependence of the trona unit cell volume up to 12.2 GPa. Black squares and red circles represent powder experimental data obtained using silicone oil and neon as PTM, respectively, while blue diamonds represent SCXRD experimental data. The solid green line corresponds to DFT calculations using the PBEsol functional. The solid black and red lines represent fits to the third-order Birch–Murnaghan equation of state for silicone oil and neon, respectively, while the violet and blue dashed lines represent fits to the second-order Birch–Murnaghan equation of state for silicone oil and neon, respectively. The inset displays the pressure dependence of the trona β angle. The estimated standard deviation (ESD) values in the experimental data are smaller than the dimensions of the symbols.

**Table 1:** Birch–Murnaghan EoS Parameters from Experimental Data Using Silicone Oil and Neon as PTMs, as well as from DFT Calculations

	2^nd^ order BM EoS		3^rd^ order BM EoS		
	*V*_0_ (Å^3^)	*K*_0_ (GPa)	*V*_0_ (Å^3^)	*K*_0_ (GPa)	*K*_p_
silicone oil	698.0(8)	43.2(11)	698.9(13)	39(4)	6(2)
neon	696.9(8)	36.1(6)	697.1(9)	35(2)	4.4(9)
DFT	-	-	694.8	35.6	3.9

The evolution of trona’s lattice parameters as a function of pressure is depicted in Figure 5. DFT calculations are in strong agreement with experimental data, particularly when neon is used as the PTM, as previously observed in Figure 4. The discrepancy between the experimental data and DFT calculations for the *a*-axis is supported by the *inset* in Figure 4, which shows the β angle as a function of pressure, reflecting the strong correlation between these parameters. In contrast, the *b*- and *c*-axes demonstrate excellent agreement between experimental data and DFT calculations. Trona’s lattice parameters and unit cell volumes at various pressures are summarized in Tables S1 and S2 for silicone oil and neon as PTM, respectively.

**Figure 5:**
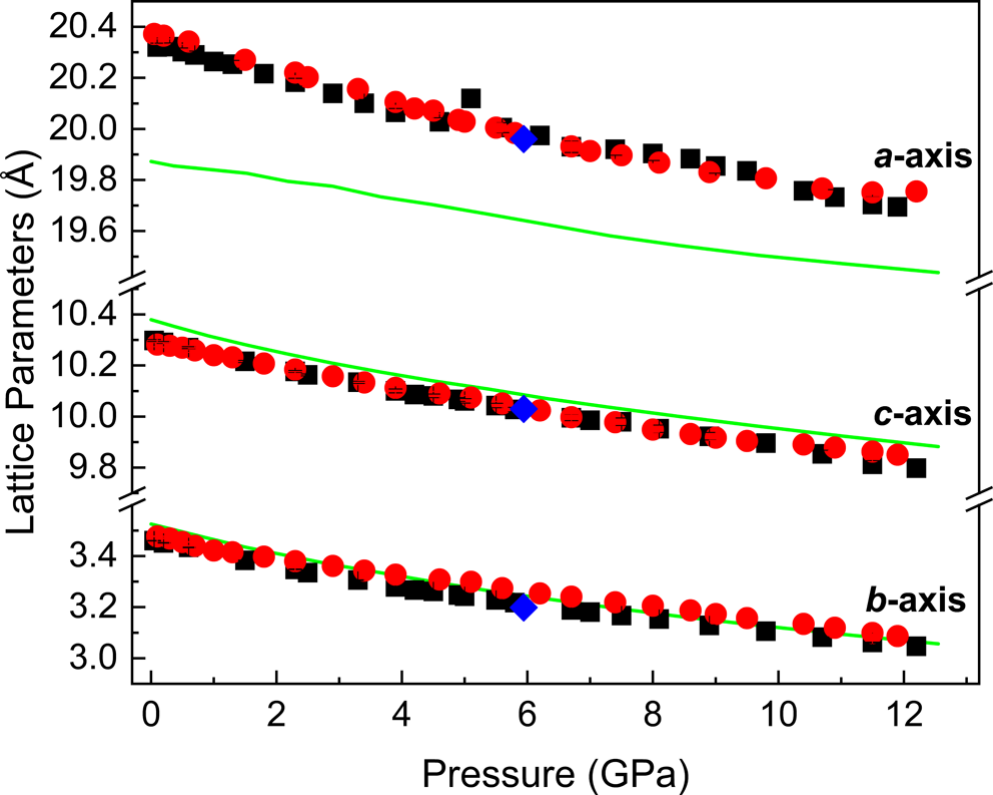
Pressure dependence of the trona lattice parameters up to 12.2 GPa. Black squares and red circles represent powder experimental data obtained using silicone oil and neon as PTM, respectively, while blue diamonds represent SCXRD experimental data. The solid green line corresponds to DFT calculations using the PBEsol functional. The estimated standard deviation (ESD) values in the experimental data are smaller than the dimensions of the symbols.

The main axes of compression and compressibility in trona were determined using PASCal v2.2.0.^50^ For silicone oil as the PTM, the main axes of compressibility are (0, 1, 0), (0.265, 0, 0.9642), and (0.8538, 0, −0.5206), with compressibilities of κ_1_ = 9.58(7) × 10^–3^ GPa^–1^, κ_2_ = 4.41(6) × 10^–3^ GPa^–1^, and κ_3_ = 2.40(13) × 10^–3^ GPa^–1^. For neon as the PTM, the main axes of compressibility are (0, 1, 0), (0.3444, 0, 0.9388), and (−0.673, 0, 0.7397), with compressibilities of κ_1_ = 10.32(9) × 10^–3^ GPa^–1^, κ_2_ = 4.74(5) × 10^–3^ GPa^–1^, and κ_3_ = 2.53(6) × 10^–3^ GPa^–1^. The *b*-axis is the most compressible, with an axial compression of ∼12% between room pressure and 12.2 GPa, while the *a*- and *c*-axes exhibit compressions of ∼3% and ∼5%, respectively. These axial compressibility values are consistent regardless of the PTM used.

The structure of trona at 5.9 GPa was determined to belong to the *I*2/*a* monoclinic space group based on experimental data. However, the structure was transformed into the *C*2/*c* standard setting while maintaining an identical topology to its room conditions counterpart. The unit cell dimensions are *a* = 19.961(1) Å, *b* = 3.199(1) Å, *c* = 10.030(3) Å, and β = 107.54(2)°, with a unit cell volume of *V* = 610.6(2) Å^3^, *Z* = 4, and a calculated density of ρ = 2.459 g·cm^–3^. Details of the XRD data collection, refinement results, and structural data obtained for trona at 5.9 GPa are provided in Tables S3, S4, and S5, where hydrogen occupies a special position, and Tables S6, S7, and S8, where hydrogen occupies a general position. Figure 6a depicts the trona structure along the *b*-axis at 5.9 GPa. Identical triple-chain units, composed of three 6-fold-coordinated edge-sharing sodium polyhedra (an octahedron flanked by two distorted trigonal prisms), are observed, as in the structure at room conditions. However, the separation between the distorted trigonal prisms (Na–Na distance) of adjacent chains decreases with increasing pressure, measuring 3.954(2) Å at room conditions and 3.7263(5) Å at 5.9 GPa.

**Figure 6:**
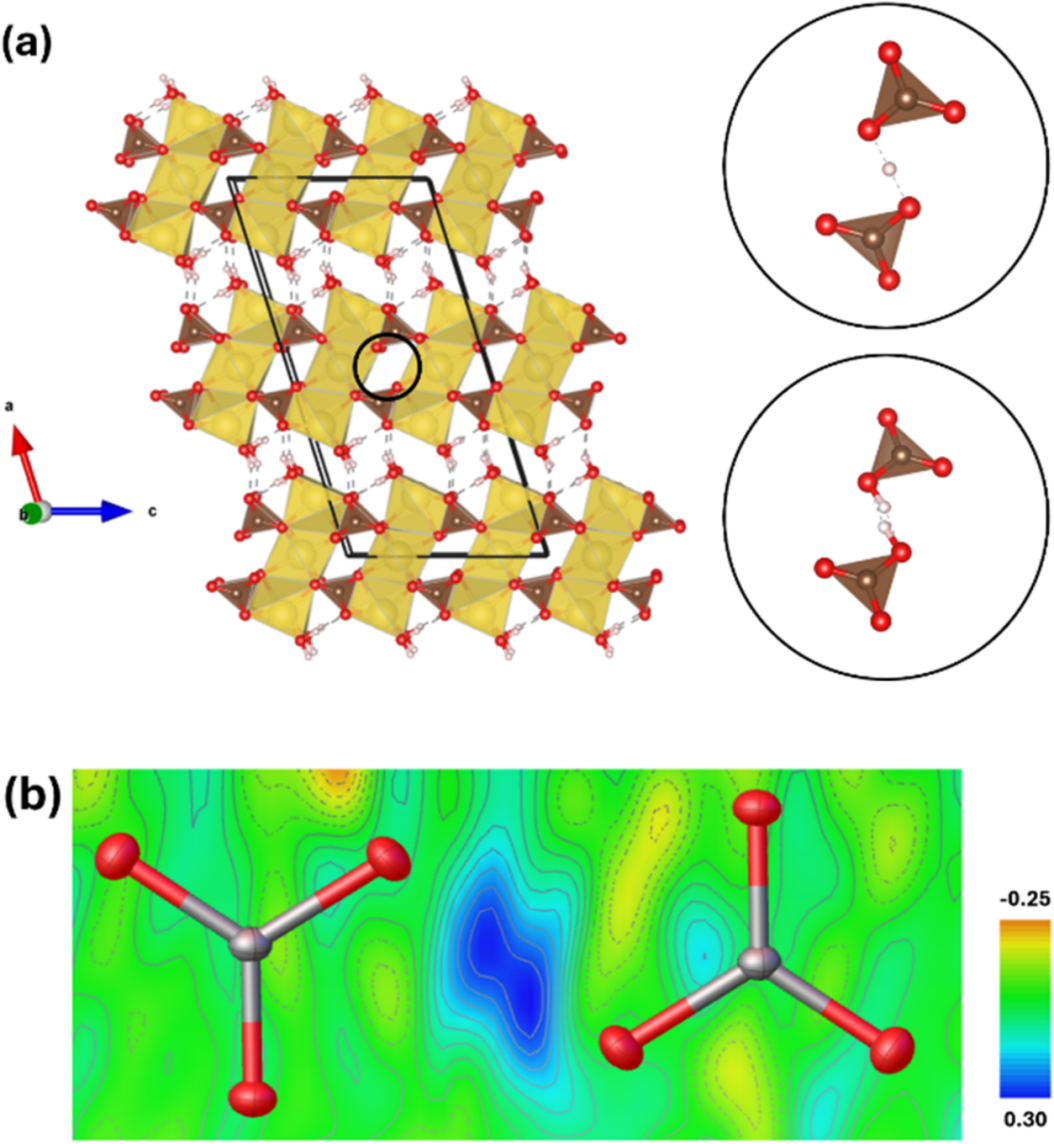
(a) Projection of Na_3_H­(CO_3_)_2_·2H_2_O trona along the *b*-axis at 5.9 GPa, with hydrogen atoms of the [HCO_3_]^−^ groups omitted in the representation. The circles indicate the hydrogen positions: the upper circle represents hydrogen in a special position (0.5, 0, 0.5), corresponding to the Wyckoff position 4b with a SoF = 1, while the lower circle represents hydrogen in a general position (0.495, 0.047, 0.485), corresponding to the Wyckoff position 8f with a SoF = 0.5 and an H–H distance of 0.65(1) Å. Sodium (Na), oxygen (O), carbon (C), and hydrogen (H) atoms are represented in yellow, red, brown, and white, respectively. (b) Difference Fourier map around two [HCO_3_]^−^ groups in trona at 5.9 GPa.

Regarding the coordination of trona at 5.9 GPa, the coordination environment of the Na atoms was assessed based on the Na–O distances and the atomic Voronoi–Dirichlet polyhedra (VDP). According to Gagné and Hawthorne,^51^ the lower and upper limits of Na–O distances for a 6-fold coordinated polyhedron at room conditions are 2.129 and 3.055 Å, respectively, while for a 7-fold coordination, these limits extend from 2.178 to 3.180 Å. Figure 7 illustrates the pressure dependence of the Na–O distances in Na2 polyhedra (corresponding to the distorted trigonal prism) in trona. Here, *d*_0_ corresponds to the average of the shortest distances (ranging from 2.220 to 2.390 Å), while *d*_1_ and *d*_2_ represent the two immediately longer distances. Finally, *d*_3_ is the longest distance observed in the distance histogram before a significant distance gap. Closed and open symbols correspond to DFT calculations and SCXRD data, respectively. At room conditions, the coordination polyhedron evaluation yields a coordination number (CN) of 6. However, the distance histogram reveals an additional Na–O distance of 3.277(2) Å, which exceeds the upper limit for 6-fold coordination according Gagné and Hawthorne and suggests the potential for higher coordination at elevated pressures. At 5.9 GPa, this additional distance reduces significantly to 2.831(3) Å, supporting the re-evaluation of the coordination number under high-pressure conditions. In distorted coordination polyhedra, it is difficult to precisely evaluate the contribution of each surrounding atom to the coordination environment. To address this, the Effective Coordination Number (ECoN) is introduced as a weighting scheme that quantifies the contribution of each neighboring atom to the coordination polyhedron, based on the distance from the central atom to each surrounding atom.^52,53^ The ECoN is defined by the following equation:

1 $$\mathrm{ECoN}=\sum_{i}w_{i}$$

where *w*_*i*_ represents the bond weight, calculated as follows:

2 $$w_{i}=\exp\left[1-{\left(l_{i}\frac{\sum_{j}\exp\left[1-{\left(\frac{l_{j}}{l_{\min}}\right)}^{6}\right]}{\sum_{j}l_{j}\exp\left[1-{\left(\frac{l_{j}}{l_{\min}}\right)}^{6}\right]}\right)}^{6}\right]$$

where *l*_*i*_ is the bond length for the *i*-th neighboring atom, *l*_*j*_ is the bond length for the *j*-th neighboring atom, and *l*_min_ is the minimum bond length in the polyhedron. As observed in the graph evolution (Figure 7), the ECoN increases gradually with pressure, while the bond distances continuously decrease.

**Figure 7:**
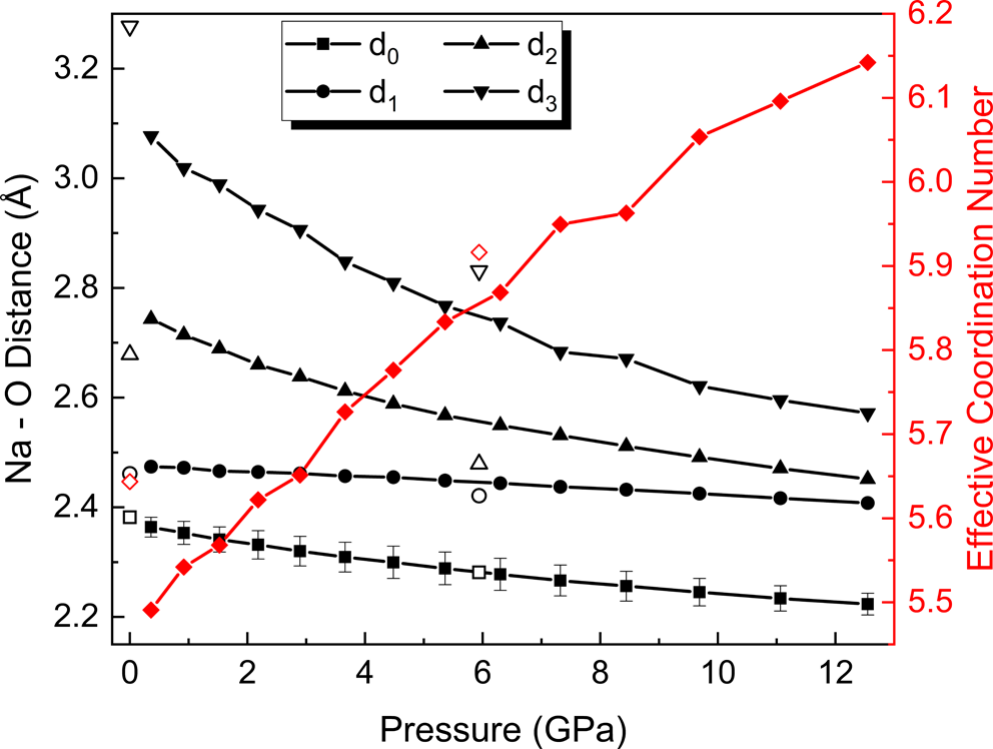
Pressure dependence of the Na–O distances in Na2 polyhedra (left *y*-axis) and the effective coordination number (right *y*-axis) up to 12.5 GPa. Black squares, circles, upward triangles, and downward triangles represent *d*_0_, *d*_1_, *d*_2_, and *d*_3_, respectively, according to DFT calculations, while red diamonds represent the effective coordination number from DFT calculations. Open symbols correspond to SCXRD experimental data at room conditions (O’Bannon et al., 2014)^9^ and 5.9 GPa (this work). The estimated standard deviation (ESD) values in the experimental data are represented by error bars of the same dimensions as the open symbols.

The VDP is a convex polyhedron whose faces are perpendicular to the lines connecting the central atom of the VDP to its neighboring atoms. Each face bisects these lines at their midpoints.^54–56^ Blatov and Serezhkin^54,57^ proposed the concepts of “major” VDP faces to identify direct neighbors (an atom directly bonded to the central atom, where the connecting line is intercepted by a VDP face). The VDP analysis for trona was conducted using ToposPro software.^56,58^ The CN was determined based on the number of major faces in each polyhedron. For trona under room conditions, the VDP contains 6 major faces, resulting in a CN = 6. At 5.9 GPa, the VDP has again 6 major faces, indicating that no apparent change in the CN occurs. However, 2 of these major faces are notably distorted, suggesting the approximation and closer interaction with an additional oxygen atom, as reflected by the additional *d*_3_ Na–O distance observed in the distance histogram (see Figure 7). Since coordination numbers are not rigidly defined and depend on how a chemical bond is characterized,^51,59^ the combination of distance and VDP analysis suggests a gradual increase in the CN with pressure. This is consistent with the closer approach of Na atoms, indicating a transition toward a CN of 7. The continuous reduction of this additional distance promotes bonding between the triple chains, forming layers along the *b*-axis.

Neutron diffraction experiments on trona, conducted by Pertlik,^15^ were compared with XRD data, revealing notable differences in the localization of hydrogen atoms. For Na atoms, the center of gravity of the atomic nucleus and the electron shell coincides almost perfectly, as expected given the spherically symmetrical electron shell of Na atoms. However, H atoms exhibited extremely strong polarization. The discrepancies between positional atomic parameters (excluding H) calculated from XRD and those obtained from neutron diffraction are almost negligible (on average, 0.005 Å), the standard deviations of the positional parameters being 0.002 Å for neutron diffraction refinements and 0.001 Å for XRD data (again, excluding H atoms). As anticipated, the most significant discrepancies were observed in the localization of H atoms, which also affected the values of O–H bond lengths. The O–H distances determined from X-ray data were systematically shorter than those obtained from neutron diffraction. This apparent shortening is attributed to the polarization of the electron shell of the H atom, with the electron density being drawn toward the donor oxygen atom. Consequently, the true error in O–H bond lengths determined via XRD data is significantly larger than the calculated standard deviations.

In all studies, the hydrogen atom forming the complex [CO_3_–H···CO_3_]^3–^ unit is dynamically disordered between two equivalent sites in a general position, corresponding to the Wyckoff position 8f. Table 2 summarizes the H–O distances and the ∠COH bond angles of the bicarbonate groups. These sites are separated by 0.211(9) Å (Choi and Mighell), 0.96(6) Å (Pertlik), and 0.87(14) Å (O’Bannon et al.), all with a partial site occupancy factor (SoF) of 0.5. In the present study, the refinement of trona at 5.9 GPa initially suggested the hydrogen atom in a special position (0.5, 0, 0.5), corresponding to the Wyckoff position 4b with a SoF = 1, as shown in the upper circle in Figure 6a. However, the difference Fourier map depicted in Figure 6b indicates distorted charge density between the two [HCO_3_]^−^ groups, suggesting disorder in the hydrogen position. This fact entails refining the hydrogen atom in a general position, as observed in the lower circle in Figure 6a, with a SoF = 0.5, separated by 0.65(1) Å, consistent with the findings of Choi and Mighell,^14^ Pertlik,^15^ and O’Bannon et al.^9^ Spahr et al.^60^ determined the structure of Li­[HC_2_O_5_] at 25(2) GPa using SCXRD, reporting the symmetrization of the hydrogen bond within the [HCO_3_]^−^ group due to pressure-induced effects. Given the low resolution of the experimental data obtained from DAC measurements, with only 44% completeness, and considering Spahr’s findings on the experimental H symmetrization, the demonstrated accuracy of DFT calculations in predicting hydrogen positions,^61^ and the structural stability with the symmetrized hydrogen observed in this work through DFT calculations, it is reasonable to consider that hydrogen occupies a special position corresponding to the Wyckoff position 4b. Once the symmetrization of the hydrogen bond is considered, the [CO_3_–H···CO_3_]^3–^ unit transforms into a sort of superanion [CO_3_···H···CO_3_]^3–^, where no distinction exists between O–H and H···O bonds, as both are equivalent when the hydrogen atom resides at an inversion center. Consequently, both interactions can be treated as hydrogen bonds exhibiting characteristics of covalent bonding (see details of the hydrogen bond characterization below).^62,63^ In our case, the Goodness of Fit (GoF) differs by less than 1% regardless of whether hydrogen occupies a special or a general position, as also observed by Choi and Mighell, indicating that the disordered H approach does not statistically improve the model.

**Table 2:** Average Covalent H–O Distances, Hydrogen Bond Lengths, and Angles for [HCO_3_]^−^ Groups in Trona Polymorphs, as Obtained from Experimental and DFT Data (This Work)

	experimental data			DFT calculations	
sample	H–O (Å)	H···O (Å)	∠DHA angle (°)	H···O (Å)	∠DHA angle (°)
Trona (Choi and Mighell, 1982)	1.13(1)^a^	1.34(1)	179(1)	1.22	112
Trona (Pertlik, 1986)	0.77(3)^a^	1.72(3)	174(4)		
Trona (O’Bannon et al., 2014)	0.86(7)^a^	1.64(7)	161(8)		
Trona at 5.9 GPa	0.92(1)^a^	1.52(1)	167(1)	1.20	109
	-	1.21(1)	109(1)		
HP phase at 14.3 GPa	-	1.36(1)	114(1)	1.20	114
HP phase at 17.9 GPa	-	1.34(1)	115(1)	1.19	116

a **Note:** Two hydrogen atoms with a partial site occupancy factor (SoF) of 0.5.

To understand the structure of hydrated carbonates an in-depth investigation into the behavior of hydrogen bonds is required.^64^ A hydrogen bond is a noncovalent interaction, typically represented as D–H···A, where D–H denotes the donor group and A the acceptor atom. It is characterized by the H···A distance and the ∠DHA angle. Generally, a hydrogen bond involves an attractive interaction occurring when the donor group exhibits moderate polarity. Using the Steiner and Jeffrey criteria for hydrogen bonds, these interactions can be further characterized.^63,65–70^ When the H atom occupies a general position, the H–O bond exhibits covalent characteristics. If the H atom is in a special position, the hydrogen bond is classified as strong with covalent character but also with high directionality. The distinction between these classifications arises from the variation in bond distances associated with different hydrogen positions, as H–O distances are shorter than H···O distances. Concerning the hydrogen bonds formed by water molecules, Table 3 summarizes the H–O distances, ∠HOH bond angles, H···O distances, and ∠DHA bond angles. These hydrogen bonds are classified as moderate, displaying an ionic character with moderate directionality.

**Table 3:** Average Covalent H–O Distances, ∠HOH Bond Angles, Hydrogen Bond Lengths, and Angles for H_2_O Groups in Trona Polymorphs, as Obtained from Experimental and DFT Data (This Work)

	experimental data				DFT calculations			
sample	H–O (Å)	∠HOH angle (°)	H···O (Å)	∠DHA angle (°)	H–O (Å)	∠HOH angle (°)	H···O (Å)	∠DHA angle (°)
Trona (Choi and Mighell, 1982)	0.97(3)	108(1)	1.80(3)	172(3)	1.00	106	1.69	173
Trona (Pertlik, 1986)	0.81(3)	107(3)	1.97(3)	172(3)				
Trona (O’Bannon et al., 2014)	0.86(4)	104(4)	1.92(3)	171(5)				
Trona at 5.9 GPa	0.92(4)	100(4)	1.77(4)	161(4)	1.00	106	1.61	174
HP phase at 14.3 GPa	0.96(9)	107(12)	1.76(9)	158(10)	1.00	103	1.62	165
HP phase at 17.9 GPa	0.94(6)	101(8)	1.77(6)	146(7)	1.00	104	1.64	157

The HP phase crystallizes within *P*1̅ triclinic space group. At 14.3 GPa the unit cell dimensions are *a* = 2.9182(2) Å, *b* = 9.328(3) Å, *c* = 10.4483(14) Å, α = 70.967(18)°, β = 83.714(9)°, and γ = 82.955(14)° with a unit cell volume of *V* = 266.10(9) Å^3^, *Z* = 2, and a calculated density of ρ = 2.821 g·cm^–3^. From the extrapolated trona EoS, the volume change across the transition is estimated to be ∼3%. Details of the XRD data collection, refinement results, and structural data obtained for the HP phase at 14.3 GPa are provided in Tables S9, S10, and S11. At 17.9 GPa the unit cell dimensions are *a* = 2.8736(3) Å, *b* = 9.219(3) Å, *c* = 10.3997(16) Å, α = 70.699(19)°, β = 83.559(11)°, and γ = 82.703(16)° with a unit cell volume of *V* = 257.19(9) Å^3^, *Z* = 2, and a calculated density of ρ = 2.919 g·cm^–3^. Details of the XRD data collection, refinement results, and structural data obtained for HP phase at 17.9 GPa are provided in Tables S12, S13, and S14, and its structure is depicted in Figure 8. The structural topology comprises three independent distorted sodium polyhedra that share either triangular faces or edges and form triple chains: two [NaO_7_] capped trigonal prismatic polyhedra and one [NaO_8_] bicapped trigonal prismatic polyhedron. Coordination numbers, determined via VDP analysis, reveal VDPs with 7, 7, and 8 major faces, respectively. The unit cell contains two triple chains of sodium polyhedra, which connect via corners to adjacent chains along the *bc* plane, forming sextuple chains as a result of the increased CN relative to the trona structure. These chains are arranged alternately in ...ABAB... sequence. Distorted [CO_3_]^2–^ groups connect the sodium polyhedral chains. Water molecules are incorporated into two of the three sodium polyhedra, resulting in [NaO_5_·3H_2_O] and [NaO_5_·2H_2_O] stoichiometries.

**Figure 8:**
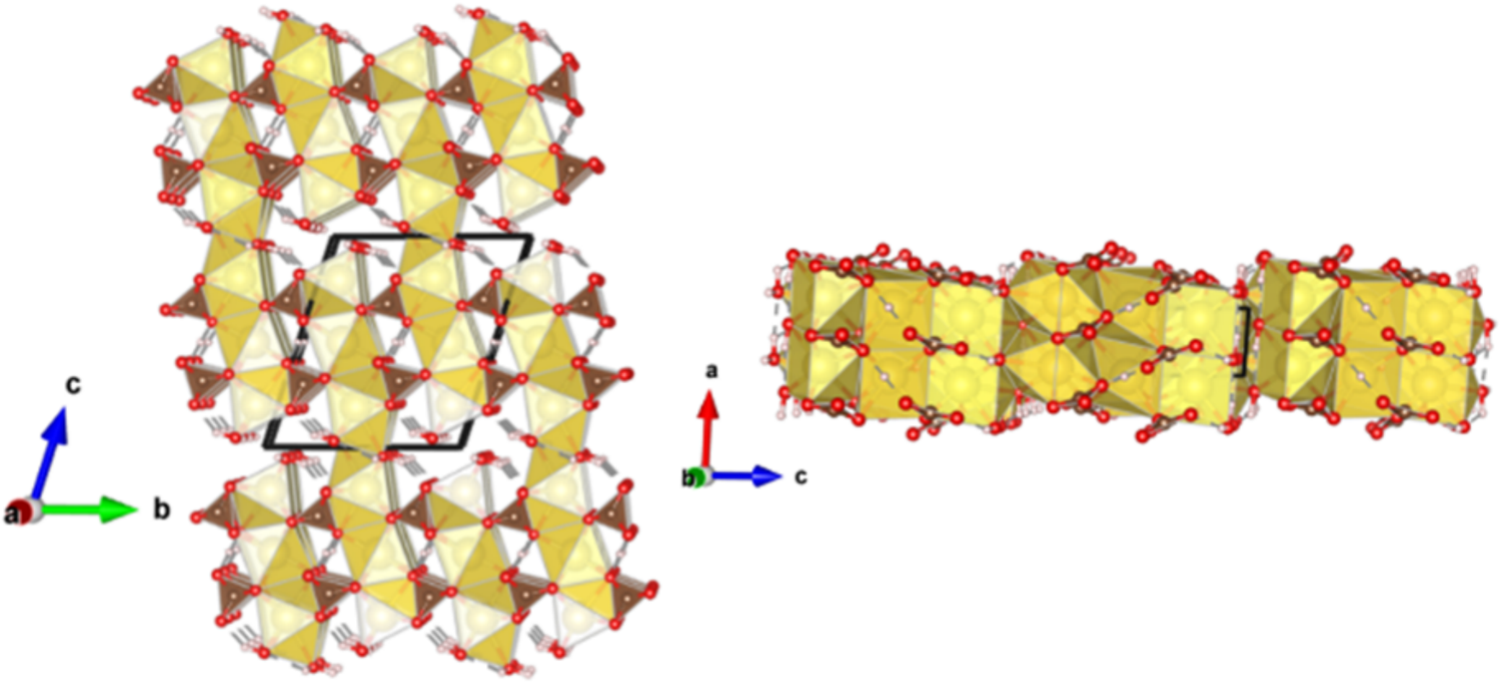
Projection of the high-pressure (HP) phase of trona along the *a*-axis (left) and *b*-axis (right) at 17.9 GPa. Sodium (Na), oxygen (O), carbon (C), and hydrogen (H) atoms are represented in yellow, red, brown, and white, respectively.

The resolution of the high-pressure data sets (completeness of 35% in both cases) was insufficient to determine hydrogen positions of [HCO_3_]^−^ group, using residual density or Fourier maps. Therefore, hydrogen positions were assigned based on the special positions discussed for trona at 5.9 GPa and obtained through DFT calculations. The hydrogen atoms were placed in special positions (0, 0.5, 0.5) and (0, 0, 0.5) - corresponding to the Wyckoff positions 1g and 1b, respectively - to maintain consistency with the chemical composition of trona.

DFT calculations do not predict the phase transition from the room monoclinic phase to the high-pressure centrosymmetric triclinic phase within the pressure range up to 24 GPa. This conclusion is supported by the energy vs volume per formula unit curves and the enthalpy vs pressure curves (*inset*) for each phase, as depicted in Figure 9. However, the HP phase becomes energetically more competitive upon compression, with an energy difference of only 0.6 mHa at 18 GPa, 1 order of magnitude smaller than the thermal energy of the atoms at room temperature. Additional DFT calculations were performed to explore the most stable triclinic phase during the transition, suggesting a noncentrosymmetric triclinic structure within the *P1* space group (HPT1) and a centrosymmetric triclinic structure within the *P*1̅ space group, with Na atoms occupying different atomic positions (HPT2). The theoretical phases, denoted as HPT1 and HPT2, were identified as energetically more stable phases above 17 and 21 GPa, respectively. Crystallographic information on these DFT calculated phases can be found in Supporting Information (Tables S15 and S16). However, it is important to emphasize that the experimental data set does not support such solutions.

**Figure 9:**
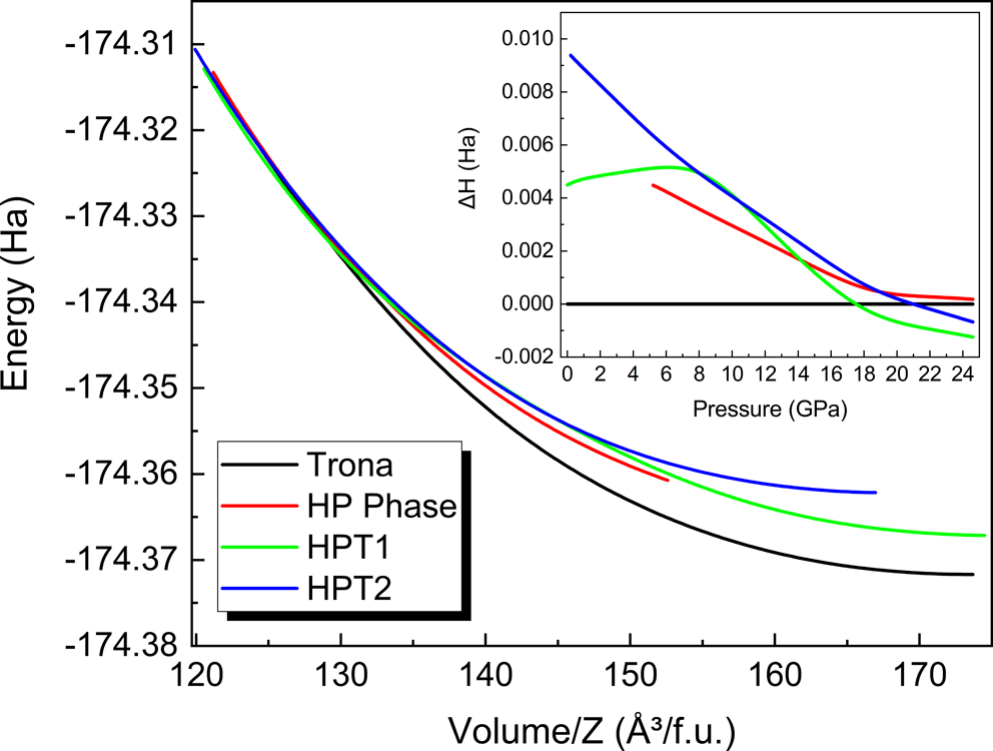
Internal energy as a function of volume per formula unit for the Na_3_H­(CO_3_)_2_·2H_2_O trona phases: trona (black line), HP phase (red line), HPT1 (green line), and HPT2 (blue line). The inset shows the enthalpy variation versus pressure curve for these polymorphs, with the trona structure taken as the reference.

## Conclusions

The structural behavior of Na_3_H­(CO_3_)_2_·2H_2_O trona mineral upon compression was studied by means of HP powder and single-crystal synchrotron XRD experiments. Our results confirm the existence of the second phase transition reported by O’Bannon et al. at 14.5 GPa from spectroscopic data,^9^ which was observed in the present work at 12.8 GPa. However, the data does not show any indication of the transition that the same authors reported at 7 GPa. The structure of the novel dense post-trona phase was determined, revealing a monoclinic to triclinic phase transition. Coordination environments are also evaluated, showing a progressive increase of the Na coordination number, with a further increase in the HP phase. The compressibility of trona was also confirmed, yielding a *K*_0_ = 35(2) GPa using the BM3 equation of state and Ne as PTM, in agreement with the *K*_0_ of 36 GPa obtained from P- and S-wave velocities by O’Bannon et al.^9^ DFT calculations confirm the phases’ stability and suggest two triclinic structures not observed experimentally.

## Supplementary Material



## Notes

Tables with experimental XRD results and structural details (PDF)
