# Supplementary material for: Compressibility and Anisotropy of Trona: Unveiling the Structure of a Dense Na3H(CO3)2·2H2O Polymorph
Source: Inorg Chem. 2025 May 15;64(20):10067–77. doi: 10.1021/acs.inorgchem.5c00642 (PMC12128025; doi:10.1021/acs.inorgchem.5c00642)
Supplement: Supplementary file 1 [file ic5c00642_si_001.pdf]

Supplementary Material

of

Compressibility and Anisotropy of Trona: Unveiling the Structure of a Dense $Na_3H(CO_3)_2 \cdot 2H_2O$ Polymorph

Benedito Donizeti Botan-Neto[1,*], David Santamaria-Perez[1,*], Lena Wedek[2], Lkhamsuren Bayarjargal[2], Ganesh Bera[1], Pablo Botella[1], Julio Pellicer-Porres[1], Alberto Otero-de-la-Roza[3], Catalin Popescu[4], and Frederico G. Alabarse[5]

[1] *Departamento de Física Aplicada-ICMUV, MALTA Consolider Team, Universitat de València, Valencia 46100, Spain*

[2] *Institute of Geosciences, Goethe University Frankfurt, Frankfurt 60438, Germany*

[3] *Departamento de Química Física y Analítica, Facultad de Química, MALTA Consolider Team, Universidad de Oviedo, Oviedo 33006, Spain*

[4] *CELLS-ALBA Synchrotron Light Facility, Cerdanyola del Vallés, 08290, Barcelona, Spain*

[5] *Elettra Sincrotrone Trieste, Trieste 34149, Italy*

***Corresponding authors' e-mails:** bebone@uv.es and david.santamaria@uv.es

Table S1 - Lattice parameters and unit cell volume of trona using silicone oil as PTM up to 11.9 GPa.

Pressure (GPa)	*a*-axis (Å)	*b*-axis (Å)	*c*-axis (Å)	β angle (°)	Volume (Å ³)
0.1(1)	20.321(15)	3.477(1)	10.282(8)	106.38(6)	697.0(5)
0.3(1)	20.322(15)	3.469(1)	10.278(8)	106.44(6)	694.9(5)
0.5(1)	20.302(15)	3.455(1)	10.270(8)	106.50(6)	690.8(5)
0.7(1)	20.290(15)	3.440(2)	10.260(8)	106.59(6)	686.4(5)
1.0(1)	20.264(15)	3.422(2)	10.240(8)	106.67(6)	680.3(5)
1.3(1)	20.253(15)	3.415(2)	10.232(8)	106.69(6)	677.9(5)
1.8(1)	20.216(15)	3.398(1)	10.208(8)	106.75(6)	671.4(5)
2.3(1)	20.183(15)	3.380(1)	10.184(8)	106.81(6)	665.2(5)
2.9(1)	20.139(15)	3.362(1)	10.158(8)	106.87(6)	658.1(5)
3.4(1)	20.101(15)	3.344(1)	10.133(8)	106.94(6)	651.5(5)
3.9(1)	20.065(15)	3.327(1)	10.112(8)	106.99(6)	645.6(5)
4.6(1)	20.028(15)	3.309(1)	10.091(8)	107.08(6)	639.2(5)
5.1(1)	20.119(19)	3.300(1)	10.075(8)	107.02(6)	639.6(5)
5.6(1)	20.005(19)	3.275(1)	10.051(8)	107.23(6)	628.9(5)
6.2(1)	19.974(19)	3.254(1)	10.024(8)	107.25(6)	622.2(5)
6.7(1)	19.930(22)	3.241(2)	9.997(9)	107.20(7)	616.9(5)
7.4(1)	19.920(24)	3.220(2)	9.978(9)	107.25(8)	610.6(6)
8.0(1)	19.904(28)	3.206(3)	9.949(11)	107.24(10)	606.3(6)
8.6(1)	19.883(28)	3.187(3)	9.932(11)	107.31(10)	600.9(6)
9.0(1)	19.854(28)	3.173(3)	9.917(11)	107.35(10)	596.3(6)
9.5(1)	19.835(28)	3.157(3)	9.905(11)	107.42(11)	591.8(6)
10.4(2)	19.758(29)	3.134(3)	9.891(11)	107.51(11)	584.1(6)
10.9(2)	19.733(29)	3.119(3)	9.878(11)	107.56(11)	579.6(6)
11.5(2)	19.704(29)	3.099(3)	9.863(11)	107.63(11)	574.0(6)
11.9(2)	19.695(30)	3.087(3)	9.851(11)	107.66(11)	570.7(6)

Table S2 - Lattice parameters and unit cell volume of trona using neon as PTM up to 12.2 GPa.

Pressure (GPa)	*a*-axis (Å)	*b*-axis (Å)	*c*-axis (Å)	β angle (°)	Volume (Å³)
0.05(5)	20.372(11)	3.460(1)	10.298(4)	106.56(3)	695.8(3)
0.2(1)	20.365(13)	3.452(1)	10.290(4)	106.62(4)	693.1(4)
0.6(1)	20.343(13)	3.434(1)	10.270(4)	106.69(4)	687.2(4)
1.5(1)	20.271(13)	3.383(1)	10.216(4)	106.88(4)	670.4(3)
2.3(1)	20.221(13)	3.348(1)	10.177(4)	107.00(4)	658.9(3)
2.5(1)	20.202(13)	3.335(1)	10.163(4)	107.05(4)	654.6(3)
3.3(1)	20.156(13)	3.306(1)	10.134(4)	107.17(4)	645.2(3)
3.9(1)	20.105(13)	3.279(1)	10.100(7)	107.25(5)	635.9(4)
4.2(1)	20.080(13)	3.267(1)	10.086(7)	107.28(5)	631.7(4)
4.5(1)	20.071(13)	3.262(1)	10.081(7)	107.30(5)	630.1(4)
4.9(1)	20.036(16)	3.248(1)	10.067(7)	107.35(5)	625.2(4)
5.0(1)	20.029(18)	3.243(2)	10.061(9)	107.36(6)	623.7(5)
5.5(1)	20.005(18)	3.228(2)	10.043(9)	107.39(6)	618.9(5)
5.8(1)	19.984(18)	3.218(2)	10.028(9)	107.41(6)	615.2(5)
6.7(1)	19.932(18)	3.189(2)	9.995(11)	107.53(8)	605.9(5)
7.0(1)	19.913(18)	3.180(2)	9.986(11)	107.55(8)	602.9(5)
7.5(1)	19.897(19)	3.167(2)	9.979(15)	107.62(8)	599.4(6)
8.1(1)	19.868(19)	3.153(2)	9.952(15)	107.61(8)	594.3(6)
8.9(1)	19.831(19)	3.129(2)	9.923(15)	107.66(8)	586.6(6)
9.8(1)	19.807(19)	3.101(2)	9.896(14)	107.74(8)	579.8(6)
10.7(1)	19.767(19)	3.082(2)	9.853(14)	107.72(8)	571.8(6)
11.5(1)	19.752(19)	3.062(2)	9.813(14)	107.70(8)	565.3(6)
12.2(1)	19.755(19)	3.047(2)	9.797(14)	107.78(8)	561.6(5)

Table S3 - Crystal data and structure refinement details for trona at 5.9 GPa.

**CCDC Number**	2417542
**Empirical Formula**	$C_2H_5Na_3O_8$
**Formula Weight [g.mol^{-1}]**	226.03
**Temperature [K]**	293
**Crystal System**	Monoclinic
**Space Group (number)**	*C2/c* (15)
**a [Å]**	19.961(1)
**b [Å]**	3.199(1)
**c [Å]**	10.030(3)
**β [°]**	107.54(2)
**Volume [Å^3]**	610.6(2)
**Z**	4
**ρ_{calc} [g.cm^{-3}]**	2.459
**μ [mm^{-1}]**	0.068
**F(000)**	456.0
**Crystal Size [μm^3]**	30 x 20 x 15
**Radiation**	Synchrotron (λ = 0.2912)
**2θ Range [°]**	5.262 to 28.084
**Index Ranges**	-10 ≤ h ≤ 9<br>-5 ≤ k ≤ 5<br>-32 ≤ l ≤ 32
**Reflections collected**	1297
**Independent reflections**	633<br>R_{int} = 0.0200<br>R_{sigma} = 0.0231
**Data/Restraints/Parameters**	633/5/66
**Goodness-of-Fit on F^2**	1.116
**Final R Indexes [I ≥ 2σ(I)]**	R_1 = 0.0378<br>wR_2 = 0.1157
**Final R Indexes [All Data]**	R_1 = 0.0435<br>wR_2 = 0.1216
**Largest Difference Peak/Hole [eÅ^{-3}]**	0.21/-0.24

Table S4 – Fractional atomic coordinates and equivalent isotropic displacement parameters for trona at 5.9 GPa (U_{eq} is defined as one-third of the trace of the orthogonalized U_{ij} tensor).

**Atom**	**x**	**y**	**z**	**U_{eq} (Å^2)**
Na1	0.0000	0.2384(2)	0.2500	0.0153(8)
Na2	0.15525(17)	0.38886(19)	-0.07170(5)	0.0182(7)
O1	0.55267(3)	0.1490(4)	-0.01653(7)	0.0168(11)
O2	0.57573(3)	0.2512(3)	0.21253(8)	0.0146(11)
O3	0.71969(3)	0.3911(3)	0.86089(7)	0.0149(10)
O4	0.65246(3)	0.4399(3)	0.10036(6)	0.0163(9)
C1	0.59367(4)	0.2829(4)	0.10437(9)	0.0109(13)
H1	0.6932(5)	0.409(7)	0.7672(17)	0.013
H2	0.5000	0.0000	0.5000	0.013
H3	0.7585(5)	0.2680(7)	0.8525(14)	0.013

Table S5 - Anisotropic displacement parameters for trona at 5.9 GPa (the anisotropic displacement factor exponent is expressed as: $-2\pi^2[h^2a^{*2}U_{11} + 2hka^*b^*U_{12} + ...]$).

Atom	U_{11} (Å^2)	U_{22} (Å^2)	U_{33} (Å^2)	U_{23} (Å^2)	U_{13} (Å^2)	U_{12} (Å^2)
Na1	0.014(3)	0.0160(4)	0.0160(5)	0.0000	0.0032(9)	0.0000
Na2	0.013(2)	0.0174(3)	0.0228(4)	-0.0010(2)	0.0017(8)	-0.0010(3)
O1	0.013(3)	0.0226(5)	0.0158(6)	0.0017(4)	0.0063(12)	-0.0018(6)
O2	0.009(3)	0.0178(4)	0.0167(6)	0.0009(3)	0.0021(13)	0.0005(7)
O3	0.005(3)	0.0224(4)	0.0174(5)	-0.0056(4)	0.0006(12)	-0.0001(6)
O4	0.016(3)	0.0194(4)	0.0132(5)	0.0022(3)	0.0019(10)	0.0019(6)
C1	0.009(4)	0.0117(4)	0.0122(6)	-0.0013(3)	0.0020(14)	0.0010(7)

Table S6 - Crystal data and structure refinement details for trona at 5.9 GPa.

**CCDC Number**	2417417
**Empirical Formula**	$C_2H_5Na_3O_8$
**Formula Weight [g.mol^{-1}]**	226.03
**Temperature [K]**	293
**Crystal System**	Monoclinic
**Space Group (number)**	*C2/c* (15)
**a [Å]**	19.961(1)
**b [Å]**	3.199(1)
**c [Å]**	10.030(3)
**β [°]**	107.54(2)
**Volume [Å^3]**	610.6(2)
**Z**	4
**ρ_{calc} [g.cm^{-3}]**	2.459
**μ [mm^{-1}]**	0.068
**F(000)**	456.0
**Crystal Size [μm^3]**	30 x 20 x 15
**Radiation**	Synchrotron (λ = 0.2912)
**2θ Range [°]**	5.262 to 28.084
**Index Ranges**	-10 ≤ h ≤ 9<br>-5 ≤ k ≤ 5<br>-32 ≤ l ≤ 32
**Reflections collected**	1300
**Independent reflections**	636<br>R_{int} = 0.0200<br>R_{sigma} = 0.0228
**Data/Restraints/Parameters**	636/5/69
**Goodness-of-Fit on F^2**	1.126
**Final R Indexes [I ≥ 2σ(I)]**	R_1 = 0.0397<br>wR_2 = 0.1216
**Final R Indexes [All Data]**	R_1 = 0.0453<br>wR_2 = 0.1276
**Largest Difference Peak/Hole [eÅ^{-3}]**	0.22/-0.29

Table S7 – Fractional atomic coordinates and equivalent isotropic displacement parameters for trona at 5.9 GPa (U_{eq} is defined as one-third of the trace of the orthogonalized U_{ij} tensor).

Atom	x	y	z	U_{eq} (Å^2)
Na1	0.0000	0.2385(2)	0.2500	0.0156(8)
Na2	0.15525(17)	0.38893(19)	-0.07165(5)	0.0186(7)
O1	0.55269(3)	0.1491(4)	-0.01661(7)	0.0175(11)
O2	0.57565(3)	0.2510(3)	0.21295(8)	0.0142(11)
O3	0.71963(3)	0.3912(3)	0.86103(7)	0.0154(10)
O4	0.65251(3)	0.4399(30	0.10021(6)	0.165(9)
C1	0.59357(4)	0.2829(4)	0.10477(9)	0.0120(13)
H1	0.6926(5)	0.407(7)	0.7686(17)	0.014
H2*	0.515(6)	0.047(16)	0.010(2)	0.014
H3	0.7596(5)	0.270(7)	0.8496(14)	0.014

***Note:** Partial site occupancy factor (SoF) of 0.5.

Table S8 - Anisotropic displacement parameters for trona at 5.9 GPa (the anisotropic displacement factor exponent is expressed as: $-2\pi^2[h^2a^{*2}U_{11} + 2hka^*b^*U_{12} + ...]$).

Atom	U_{11} (Å²)	U_{22} (Å²)	U_{33} (Å²)	U_{23} (Å²)	U_{13} (Å²)	U_{12} (Å²)
Na1	0.014(3)	0.0161(4)	0.0163(5)	0.0000	0.0035(9)	0.0000
Na2	0.014(2)	0.0176(3)	0.0232(4)	-0.0011(2)	0.0023(8)	-0.0010(3)
O1	0.016(3)	0.0227(5)	0.0158(6)	0.0018(4)	0.0068(12)	-0.0016(6)
O2	0.008(4)	0.0181(4)	0.0167(6)	0.0011(3)	0.0014(13)	0.0010(6)
O3	0.006(3)	0.0224(4)	0.0177(6)	-0.0056(3)	0.0006(12)	-0.0001(6)
O4	0.016(3)	0.0196(4)	0.0133(5)	0.0022(3)	0.0018(10)	0.0019(5)
C1	0.013(4)	0.0117(4)	0.0123(6)	-0.0012(3)	0.0029(14)	0.0010(7)

Table S9 - Crystal data and structure refinement details for the HP phase at 14.3 GPa.

**CCDC Number**	2417457
**Empirical Formula**	$C_2H_5Na_3O_8$
**Formula Weight [g.mol^{-1}]**	226.03
**Temperature [K]**	293
**Crystal System**	Triclinic
**Space Group (number)**	$P\bar{1}$ (2)
**a [Å]**	2.9182(2)
**b [Å]**	9.328(3)
**c [Å]**	10.4483(14)
**α [°]**	70.967(18)
**β [°]**	83.714(9)
**γ [°]**	82.955(14)
**Volume [Å^3]**	266.10(9)
**Z**	2
**ρ_{calc} [g.cm^{-3}]**	2.821
**μ [mm^{-1}]**	0.078
**F(000)**	228.0
**Crystal Size [μm^3]**	30 x 20 x 15
**Radiation**	Synchrotron (λ = 0.2912)
**2θ Range [°]**	5.084 to 28.062
**Index Ranges**	$-4 \le h \le 4$<br>$-6 \le k \le 8$<br>$-16 \le l \le 17$
**Reflections collected**	1362
**Independent reflections**	861<br>R_{int} = 0.0359<br>R_{sigma} = 0.0538
**Data/Restraints/Parameters**	861/6/130
**Goodness-of-Fit on F^2**	1.063
**Final R Indexes [I ≥ 2σ(I)]**	R_1 = 0.0746<br>wR_2 = 0.2292
**Final R Indexes [All Data]**	R_1 = 0.1124<br>wR_2 = 0.2725
**Largest Difference Peak/Hole [eÅ^{-3}]**	0.48/-0.45

Table S10 – Fractional atomic coordinates and equivalent isotropic displacement parameters for for the HP phase at 14.3 GPa (U_{eq} is defined as one-third of the trace of the orthogonalized U_{ij} tensor).

Atom	x	y	z	U_{eq} (Å^2)
Na2	0.1542(7)	-0.1055(6)	0.2063(2)	0.026(2)
Na3	0.9864(6)	0.2281(5)	0.50130(19)	0.026(2)
Na1	0.1272(7)	0.4081(6)	0.1981(3)	0.023(2)
C1	0.6876(16)	0.6031(17)	0.3195(5)	0.031(5)
O1	0.6018(13)	0.0896(10)	0.1845(4)	0.019(3)
OW2	-0.2530(13)	0.3693(11)	0.0407(4)	0.028(4)
O4	0.8679(11)	0.4708(9)	0.4035(3)	0.015(3)
O6	0.3868(12)	-0.0152(10)	0.3987(4)	0.024(4)
OW1	-0.2418(13)	-0.1372(12)	0.0520(4)	0.033(4)
C2	0.5310(18)	0.1062(18)	0.3024(5)	0.045(6)
O3	0.5606(12)	0.5900(10)	0.2085(4)	0.027(4)
O2	0.5989(15)	0.2156(13)	0.3299(4)	0.026(4)
O5	0.6370(14)	0.7181(15)	0.3481(5)	0.039(5)
HW1A	-0.2300(400)	-0.2470(60)	0.0820(90)	0.047
HW1B	-0.4300(400)	-0.1040(180)	-0.0240(100)	0.047
HW2A	-0.4200(400)	0.3900(200)	-0.0360(80)	0.047
HW2B	-0.3100(400)	0.2780(130)	0.1130(100)	0.047
H	0.0000	0.5000	0.5000	0.047
HA	0.0000	0.0000	0.5000	0.047

Table S11 - Anisotropic displacement parameters for the HP phase at 14.3 GPa (the anisotropic displacement factor exponent is expressed as: $-2\pi^2[h^2a^{*2}U_{11} + 2hka^*b^*U_{12} + \ldots])$.

Atom	U_{11} (Å^2)	U_{22} (Å^2)	U_{33} (Å^2)	U_{23} (Å^2)	U_{13} (Å^2)	U_{12} (Å^2)
Na2	0.0181(9)	0.038(6)	0.0190(9)	-0.0027(17)	-0.0036(7)	-0.0060(13)
Na3	0.0201(10)	0.043(6)	0.0122(9)	-0.0038(16)	-0.0019(7)	-0.0074(14)
Na1	0.0173(6)	0.031(5)	0.0212(10)	-0.0080(16)	-0.0029(7)	-0.0009(12)
C1	0.0097(16)	0.062(14)	0.0097(18)	0.004(4)	-0.0004(13)	0.002(3)
O1	0.0270(16)	0.022(9)	0.0104(12)	-0.008(2)	-0.0026(11)	-0.005(2)
OW2	0.0218(15)	0.043(10)	0.0135(13)	0.000(3)	-0.0018(12)	-0.004(2)
O4	0.0218(14)	0.014(8)	0.0096(11)	-0.004(2)	-0.0028(10)	-0.0023(19)
O6	0.0242(15)	0.036(9)	0.0108(12)	-0.006(3)	0.0013(11)	-0.004(2)
OW1	0.0233(16)	0.054(10)	0.0130(13)	0.004(3)	-0.0024(12)	-0.008(2)
C2	0.0107(16)	0.093(16)	0.0128(16)	0.014(4)	-0.0042(13)	-0.015(3)
O3	0.0197(14)	0.049(9)	0.0109(12)	0.000(3)	-0.0043(10)	-0.009(2)
O2	0.0172(16)	0.042(11)	0.0140(14)	0.001(3)	-0.0031(12)	-0.009(2)
O5	0.0157(16)	0.074(14)	0.0126(16)	0.005(4)	0.0012(12)	-0.001(3)

Table S12 - Crystal data and structure refinement details for the HP phase at 17.9 GPa.

**CCDC Number**	2417458
**Empirical Formula**	$C_2H_5Na_3O_8$
**Formula Weight [g.mol^{-1}]**	226.03
**Temperature [K]**	293
**Crystal System**	Triclinic
**Space Group (number)**	$P\bar{1}$ (2)
**a [Å]**	2.8736(3)
**b [Å]**	9.219(3)
**c [Å]**	10.3997(16)
**α [°]**	70.699(19)
**β [°]**	83.559(11)
**γ [°]**	82.703(16)
**Volume [Å^3]**	257.19(9)
**Z**	2
**ρ_{calc} [g.cm^{-3}]**	2.919
**μ [mm^{-1}]**	0.081
**F(000)**	228.0
**Crystal Size [μm^3]**	30 x 20 x 15
**Radiation**	Synchrotron (λ = 0.2912)
**2θ Range [°]**	5.116 to 28.072
**Index Ranges**	$-4 \leq h \leq 4$<br>$-6 \leq k \leq 8$<br>$-16 \leq l \leq 17$
**Reflections collected**	1258
**Independent reflections**	786<br>R_{int} = 0.0146<br>R_{sigma} = 0.0273
**Data/Restraints/Parameters**	786/9/130
**Goodness-of-Fit on F^2**	1.106
**Final R Indexes [I ≥ 2σ(I)]**	R_1 = 0.0765<br>wR_2 = 0.2111
**Final R Indexes [All Data]**	R_1 = 0.1043<br>wR_2 = 0.2400
**Largest Difference Peak/Hole [eÅ^{-3}]**	0.44/-0.44

Table S13 – Fractional atomic coordinates and equivalent isotropic displacement parameters for for the HP phase at 17.9 GPa (U_{eq} is defined as one-third of the trace of the orthogonalized U_{ij} tensor).

Atom	x	y	z	U_{eq} (Å^2)
Na1	0.8734(6)	0.5902(5)	0.8035(2)	0.0284(19)
Na2	0.0068(5)	0.7721(5)	0.49920(16)	0.0237(18)
Na3	0.8479(6)	0.1052(5)	0.7942(2)	0.0329(19)
O1	0.4441(10)	0.4090(8)	0.7925(3)	0.029(3)
O3	0.3639(12)	0.2794(12)	0.6519(4)	0.040(4)
O4	0.4046(11)	0.9074(9)	0.8168(3)	0.030(3)
O6	0.1342(10)	0.5308(8)	0.5960(3)	0.026(3)
OWB	1.2373(11)	0.1363(9)	0.9484(3)	0.023(3)
OWA	1.2452(11)	0.6308(9)	0.9622(3)	0.024(3)
O2	0.6199(10)	1.0108(9)	0.6014(3)	0.033(3)
O5	0.3962(12)	0.7840(12)	0.6705(3)	0.037(4)
C1	0.4754(16)	0.8878(16)	0.6988(4)	0.040(5)
C2	0.3210(15)	0.4020(16)	0.6803(4)	0.035(5)
HWAA	1.2300(300)	0.7390(60)	0.9360(80)	0.042
HWBA	1.5770(170)	0.1080(140)	0.9580(70)	0.042
HWAB	1.5680(160)	0.6050(60)	0.9520(70)	0.042
HWBB	1.2400(300)	0.2430(60)	0.9250(70)	0.042
H6	0.0000	0.5000	0.5000	0.042
H	0.0000	0.0000	0.5000	0.042

Table S14 - Anisotropic displacement parameters for the HP phase at 17.9 GPa (the anisotropic displacement factor exponent is expressed as: $-2\pi^2[h^2a^{*2}U_{11} + 2hka^*b^*U_{12} + \ldots])$.

Atom	U_{11} (Å^2)	U_{22} (Å^2)	U_{33} (Å^2)	U_{23} (Å^2)	U_{13} (Å^2)	U_{12} (Å^2)
Na1	0.0175(8)	0.045(5)	0.0189(8)	-0.0027(14)	-0.0033(6)	-0.0052(11)
Na2	0.0200(8)	0.035(5)	0.0129(7)	-0.0022(13)	-0.0034(6)	-0.0033(11)
Na3	0.0193(8)	0.056(5)	0.0176(7)	-0.0017(15)	-0.0044(6)	-0.0078(12)
O1	0.0196(11)	0.047(7)	0.0119(10)	0.005(2)	-0.0063(9)	-0.0070(18)
O3	0.0195(14)	0.069(11)	0.0147(13)	0.011(3)	-0.0036(11)	-0.008(2)
O4	0.0257(13)	0.048(8)	0.0109(9)	-0.002(2)	-0.0019(9)	-0.004(2)
O6	0.0215(12)	0.038(7)	0.0115(10)	0.003(2)	-0.0057(9)	-0.0024(17)
OWB	0.0251(13)	0.032(8)	0.0126(10)	-0.005(2)	-0.0044(10)	-0.007(2)
OWA	0.0243(13)	0.032(8)	0.0142(11)	-0.002(2)	-0.0034(10)	-0.009(2)
O2	0.0219(12)	0.057(8)	0.0116(11)	0.001(2)	-0.0006(10)	-0.0017(19)
O5	0.0154(13)	0.069(10)	0.0141(12)	0.004(3)	-0.0058(10)	-0.004(2)
C1	0.0141(15)	0.080(13)	0.0111(13)	0.009(3)	-0.0052(11)	-0.0010(3)
C2	0.0155(15)	0.067(13)	0.0099(14)	0.005(3)	-0.0022(12)	-0.009(3)

Table S15 – Fractional atomic coordinates for the DFT-predicted high-pressure phase (HPT1) at 17.6 GPa. The phase has lattice parameters a = 2.8498 Å, b = 9.2979 Å, c = 10.3791 Å, α = 70.0857°, β = 82.7529°, and γ = 82.5411° with a unit cell volume of V = 255.39 Å^3, Z = 2.

Atom	x	y	z
Na1	0.1378	0.8978	0.2020
Na2	0.8326	0.1020	0.7984
Na3	0.9808	0.2261	0.5013
Na4	0.9922	0.7736	0.4993
Na5	0.1140	0.4125	0.1947
Na6	0.8593	0.5870	0.8059
C1	0.6709	0.6003	0.3202
C2	0.3017	0.3993	0.6805
C3	0.5218	0.1079	0.3008
C4	0.4507	0.8917	0.7001
O1	0.5919	0.0972	0.1815
O2	0.3770	0.9024	0.8194
O3	0.7381	0.3698	0.0365
O4	0.2351	0.6295	0.9642
O5	0.8542	0.4742	0.4057
O6	0.1187	0.5256	0.5951
O7	0.3675	0.9881	0.3996
O8	0.6069	0.0113	0.6016
O9	0.7451	0.8610	0.0505
O10	0.2249	0.1385	0.9501
O11	0.5450	0.5957	0.2080
O12	0.4281	0.4037	0.7927
O13	0.5953	0.2248	0.3288
O14	0.3786	0.7747	0.6718
O15	0.6244	0.7242	0.3482
O16	0.3474	0.2755	0.6523
H1	0.6884	0.7502	0.1014
H2	0.2816	0.2493	0.8995
H3	0.5441	0.8906	0.9739
H4	0.4289	0.1084	0.0260
H5	0.4773	0.3935	0.9770
H6	0.4961	0.6060	0.0236
H7	0.7439	0.2547	0.0811
H8	0.2281	0.7446	0.9197
H9	0.9863	0.4999	0.5004
H10	0.4873	0.9998	0.5004

Table S16 – Fractional atomic coordinates for the DFT-predicted high-pressure phase (HPT2) at 20.7 GPa. The phase has lattice parameters *a* = 2.7609 Å, *b* = 9.0786 Å, *c* = 10.8021 Å, α = 69.0329°, β = 79.5057°, and γ = 80.9663° with a unit cell volume of V = 247.3548 $Å^3$, Z = 2.

Atom	x	y	z
Na1	0.8937	0.5963	0.8042
Na2	0.1069	0.4041	0.1954
Na3	0.0012	0.7501	0.4997
C1	0.4818	0.8997	0.6916
C2	0.5197	0.1008	0.3077
O1	0.4949	0.3995	0.8084
O2	0.5054	0.6011	0.1910
O3	0.3827	0.2816	0.6677
O4	0.6186	0.7188	0.3317
O5	0.5733	0.5299	0.5925
O6	0.4299	0.4703	0.4068
O7	0.2355	0.1335	0.9586
O8	0.7646	0.8672	0.0409
H1	0.2693	0.7501	0.9082
H2	0.7312	0.2507	0.0912
H3	0.5031	0.1050	0.0142
H4	0.4964	0.8959	0.9855
H5	0.5019	0.0001	0.4997
